# Mineralisation of tubular bones is affected differently by low phosphorus supply in growing‐finishing pigs

**DOI:** 10.1002/jsfa.9583

**Published:** 2019-03-21

**Authors:** Kristina U Sørensen, Juan M Shiguetomi‐Medina, Hanne D Poulsen

**Affiliations:** ^1^ Department of Animal Science, Faculty of Science and Technology Aarhus University Tjele Denmark; ^2^ Orthopaedic Research Laboratory, Faculty of Health Sciences Aarhus University Aarhus Denmark

**Keywords:** bone, mineralisation, phosphorus, pigs

## Abstract

**BACKGROUND:**

Phosphorus (P) supply is essential for bone mineralisation. Reduced P may result in osteopenia, whereas excessive P may result in environmental impacts. The objective was to study the long‐term effect of three dietary P levels on net bone mineralisation in growing‐finishing pigs. Eighteen female pigs were fed low P (LP (4.1)), medium P (MP (6.2)) or high P (HP (8.9 g P kg^−1^ DM)) from 39.7 until 110 kg. Trabecular, cortical and overall bone mineral density (BMD), ash, calcium (Ca) and P were determined after slaughter.

**RESULTS:**

The LP diet generally reduced the BMD, ash, Ca and P in all bones, though all measures were markedly lowered in femur compared with humerus. The trabecular BMD in LP pigs was only different in the distal section compared to the MP‐fed pigs (*P* < 0.05). In addition, ash, Ca and P were lower in the proximal and distal sections. No significant effect of HP was seen. Conclusively, LP caused lower net bone mineralisation, mainly of femur. The trabecular tissue of the distal bones seems to be most metabolically active.

**CONCLUSIONS:**

The MP level was sufficient for net bone mineralisation. Femur is recommended for studying bone fragility whereas humerus seems useful to study increased P retention. © 2019 The Authors. *Journal of the Science of Food and Agriculture* published by John Wiley & Sons Ltd on behalf of Society of Chemical Industry.

## INTRODUCTION

Dietary phosphorus (P) supply to pig diets is of great interest because intensive animal production can lead to massive environmental problems and, furthermore, attention is also directed towards the limited global P resources. Improvements in utilisation of P in feedstuffs for pigs are urgently needed in order to reduce the P supply and excretion. However, the P resource and environmental issues have to be solved without compromising pig health and welfare caused by imbalance in the P and calcium (Ca) metabolism created by too low dietary P supply.^1^

The regulation of Ca and P metabolism is a complex interplay between parathyroid hormone, calcitonin and 1.25‐dihydroxycholecalciferol controlling the flow between the intestine, bones and kidney.^2^ Phosphorus is absorbed in the intestine by paracellular and transcellular mechanisms^3^ and the kidneys mediate the excretion of P being the main regulator of P homeostasis.^4^ Contrarily, Ca is regulated through the absorptive processes in the intestine as well as reabsorption in the kidneys mediated by parathyroid hormone.^5^, ^6^ The action of the hormonal system maintains an adequate blood concentration to allow mineralisation of the bones. Appropriate mineral supply is essential for the bone structure to become sufficiently strong and to ensure optimal bone expansion and bone mass accumulation during growth. In particular, a continuous dietary P and Ca supply is important for the formation of hydroxyapatite.^7^ However, the most functionally important property of a bone is not exclusively to increase its mass by maximising mineral accumulation, but rather to make the bones functionally strong through the interplay between sufficient mineral accumulation and bone architecture development.^8^, ^9^

Leg problems in young pigs are often attributed to osteopenia resulting from dietary P and Ca deficiency.^10^, ^11^, ^12^ Calcium has traditionally drawn the primary attention concerning net mineralisation of the skeleton. However, also dietary P deficiency has been shown to cause reduced net mineralisation of tubular bones in growing pigs, even if dietary Ca was supplied at recommended levels.^13^, ^14^ In contrast, high dietary P (18 and 12 *versus* 6 g kg^−1^ (control)) was found to inhibit longitudinal bone growth and to impair bone mineral density (BMD) in the femur of growing rats.^15^ However, growing pigs fed excess Ca and P (excess Ca: 13.0 g d^−1^ *versus* 8.4 g d^−1^; excess P: 11.7 g d^−1^ *versus* 7.7 g d^−1^) showed increased overall bone mineral accretion and reduced bone resorption.^16^ Furthermore, a positive correlation between increased P intake and BMD was reported in children.^17^ Studies of the length and fresh weight of the tibia–fibula and femur in growing pigs revealed no response to low dietary P, whereas low weight of the defatted bones was reported implying poor net mineralisation.^14^ A deeper understanding of the influence of P intake on bone metabolism is required for determination of more exact P feeding recommendations for pigs considering at the same time environmental concerns and animal welfare and productivity.

The aim of the work reported here was to examine whether the net mineralisation of the weight‐bearing bones and the trabecular or cortical bone tissues were affected in terms of BMD, bone ash and bone mineral content, when the dietary P supply – as a single factor – was below, at or above the feeding recommendations during the growing period of pigs (45–110 kg BW). The overall aim was to bring about safe possibilities for improved P utilisation and reduced P emissions in pig farming.

## MATERIALS AND METHODS

### Animals, diets and housing

Eighteen female crossbred pigs ((Landrace × Yorkshire) × Duroc) from six litters of three pigs were included in the experiment. Littermates (39.7 kg) were randomised to three treatment groups: low P (LP), medium P (MP) and high P (HP) (Table 1). A basal diet containing barley, wheat and soybean meal was formulated according to the current Danish recommendations for growing‐finishing pigs (45–105 kg BW)^18^ except for the content of P and Ca (Table 2). In general, the Danish recommendations are similar to or slightly higher than the recommendations given by NRC.^19^ The Danish recommendations are given as apparent total tract digestibility and not standardised total tract digestibility (STTD) or standardised ileal digestibility of P as the endogenous P loss was not determined experimentally. The determination of endogenous P loss would require a P‐free diet. Still, the STTD of P can be calculated as the endogenous P loss is assumed to be constant according to NRC.^19^ However, we suppose that the change to STTD or standardised ileal digestibility of P will not affect the overall conclusions. The basal diet (Table 2) was produced as one batch and steam‐pelleted at 90 °C to deactivate the plant phytase. The pellets were crumbled and divided into three equal portions. Monocalcium phosphate and calcium carbonate (CaCO_3_) were supplemented to each of the three parts to meet the planned content of the three diets (Table 1). The pigs were individually weighed once a week to adjust the daily feed allowance to the actual growth rate. The pigs were fed twice per day in two equal meals. Throughout the experiment, the pigs were housed individually in pens with free access to water. For further information about animal performance, feed, etc., see Sørensen *et al.* 20

**Table 1:** Planned and analysed composition of experimental diet

	Diet^a^		
	LP	MP	HP
Planned:			
Total P, g kg^−1^ DM^b^	4.2	6.5	8.6
Total Ca, g kg^−1^ DM	8.7	8.7	8.7
Analysed:			
Dry matter (DM), g kg^−1^	906	906	910
Total P, g kg^−1^ DM	4.1	6.2	8.9
Total Ca, g kg^−1^DM	7.4	8.1	9.0
Phytate P, g kg^−1^ DM	2.7	2.7	2.7
Phytase, FTU kg^−1^	ND^c^	ND^c^	ND^c^
ATTD^d^ P, g kg^−1^ DM^e^	1.5	3.1	4.9

a P, low‐phosphorus diet; MP, medium‐phosphorus diet; HP, high‐phosphorus diet.

b DM, dry matter.

c ND, not detectable (below 50 FTU kg^−1^ DM).

d ATTD, apparent total tract digestibility.

e Sørensen *et al.* 20

**Table 2:** Ingredients and chemical composition of experimental basal diet

	Basal diet
Ingredients, g kg^−1^	
Barley	550.0
Wheat	220.8
Soybean meal	180.8
Animal fat	20.0
Molasses	10.0
Calcium carbonate	9.0
Sodium chloride	5.4
Vitamin/mineral premix^a^	2.0
Amino acids	2.0
Chemical composition, g kg^−1^	
Dry matter (DM)	858
Ash	44
Fat	43
Protein	162
Gross energy, MJ kg^−1^	16.1

a Providing per kg diet: 84 mg iron; 100 mg zinc; 42 mg manganese; 15 mg copper; 0.21 mg iodine; 0.3 mg selenium; 76.4 mg d,l‐*α*‐tocopherol; 2.1 mg vitamin K_3_; 2.1 mg vitamin B_1_; 2.1 mg vitamin B_2_; 3.15 mg vitamin B_6_; 10.5 mg d‐pantotenic acid; 21 mg niacin; 0.05 mg biotin; 0.02 mg vitamin B_12_; 4200 IU vitamin A; 420 IU vitamin D_3_.

### Sample collection and bone processing

At the completion of the experiment, all pigs were slaughtered. The left humerus, femur, IV metacarpal and IV metatarsal were removed and stored at −20 °C. Prior to analysis, the bones were thawed at 4 °C, and all extraneous tissues were manually removed. For determination of ash, Ca and P, the humerus and femur were divided into three sections: proximal, medial and distal. The proximal and distal sections were removed from the diaphysis at 0.25 of the full length of the bone and were measured from either the proximal or the distal ends. The samples were manually broken into minor pieces and placed individually in an autoclave at 120 °C for 4 h, after which they were cleaned of remaining extraneous tissue, further crushed and defatted in technical acetone for 2 h. The acetone was discarded, and the samples were left for evaporation for 2 days followed by 1 h at 80 °C for complete evaporation of acetone. The dry, defatted samples were weighed and milled using a knife mill, and the resulting bone powder was stored at −20 °C until analysis.

### Incineration and chemical analysis of diets

All experimental diets were analysed for phytate P and phytase activity according to Poulsen *et al.* 21 The ash content was determined by incineration at 525 °C for 6 h. Additionally, the diets were analysed for total P according to Poulsen *et al.* 21 Dry matter was assessed in the diets by oven drying at 103 °C for 20 h. All analyses were performed in duplicate.

### Bone mineral density

The BMD was measured at different segments of the bone: in the diaphysis (femur and humerus; cross‐sections at 0.50 of the length) and at 0.10 of the bone length from either the proximal or the distal ends (metatarsal and the metacarpal; entire bone). The BMD were measured by use of quantitative computed tomography (QCT; Stratec XCT 2000 bone scanner, Stratec Medizintechnik GmbH, Pforzheim, Germany). The QCT method determines the amount of X‐ray electromagnetic radiation that the bone sample absorbs and this technique allows separate determinations of volumetric BMD of the cortical and trabecular bone compartments as well as the area‐weighted BMD (overall BMD), providing insight into the effects of diet on the bone structure and density.^22^, ^23^

### Calcium and phosphorus in bones

The bone tissue samples (2 g) were ashed at 450 °C for 4 h, acidified with 10 mL of nitric acid (210 g kg^−1^) and transferred to a 50 mL flask with Milli‐Q water. These samples were diluted (1:4000) with 10 g kg^−1^ nitric acid before analysis, and all samples were prepared in duplicate. Calcium and P content of the milled bone tissue was determined by use of inductively coupled plasma mass spectrometry using an X series^II^ instrument equipped with a conventional Mainhard nebuliser and a Peltier cooled quartz impact bead spray chamber operated at 3 °C (Thermo Electron Cooperation, Bremen, Germany) set with a CETAC auto sampler model ASX‐520. The instrument settings were: forward power, 1400 W; plasma gas (Ar), 13 L min^−1^; nebuliser gas (Ar), 0.9 L min^−1^; auxiliary gas (Ar), 0.7 L min^−1^. The sample uptake was approximately 0.4 mL min^−1^ and ^45^Sc, ^71^Ga and ^103^Rh were used as internal standards with interpolation. Data were collected using PlasmaLab version 2.5.9.30 (Thermo Electron).

### Statistical analyses

The data were analysed statistically by use of the MIXED procedure of SAS version 9.2^24^ according to the following model:

$$Y_{\mathrm{ijk}}=\mu +\alpha_{i}+\beta_{j}+\epsilon_{\mathrm{ijk}}$$

where *Y*_*ijk*_ is the dependent variable, *μ* is the overall mean, *α*_*i*_ is the fixed effect of diet (*i* = 1,2,3), *β*_*j*_ is the random effect of litter and *ϵ*_*ijk*_ is the residual error ∼ *N*(0, *σ*_*i*_^2^). The normality of the residuals was tested. The results are presented as least square means (LSmeans), and the variance is presented as the standard error of the LSmeans (SEM). Pairwise comparisons of the LSmeans were made using the PDIFF option in SAS. Differences were considered significant when *P* < 0.05.

## RESULTS

All animal experimental procedures were carried out in accordance with the Danish Ministry of Justice, Law no. 253 of 8 March 2013 concerning experiments with animals and care of experimental animals, and licence issued by the Danish Animal Experiments Inspectorate (DAEI), Ministry of Food, Agriculture and Fisheries, Danish Veterinary and Food Administration. The protocols were approved by DAEI before initiation of the experiment.

### Chemical composition of diet

The analysed P content of the diet was as expected (Table 1); however, the chemical analysis of Ca (planned value: 8.7 g kg^−1^ DM in all three diets) showed deviations from the planned content (3–15%). The ratio between dietary Ca and P (Ca:P) corresponded to the planned values. The phytate P content was similar for all three diets and corresponded to 0.65 of the intrinsic P content. Furthermore, the phytase activity was below the detection limit (<50 FTU kg^−1^), as anticipated due to the heat treatment.

### Bone mineral density

The experiment was accomplished successfully, and all pigs completed the experiment. In general, the BMD of all bones was significantly lower in the LP‐fed pigs than in the MP‐ and HP‐fed pigs (Table 3). The overall femur BMD was 39% lower in the LP pigs compared to the other groups, whereas the overall humerus BMD was 23–27% lower in the LP pigs compared to the other groups. The overall BMD of all three sections of the metacarpal and metatarsal was significantly lower in pigs fed the LP diet compared with the other diets (*P* < 0.001) but to a lesser degree than observed in the femur. The cortical BMD of the medial bone sections was generally lower in pigs fed the LP diet than in the two other groups (*P* < 0.001). Furthermore, the LP supply resulted in lower trabecular BMD in the distal metacarpal and metatarsal compared with the other groups (*P* < 0.05), whereas the trabecular BMD in the proximal metacarpal was not affected by the LP diet. Generally, BMD results were similar in pigs fed the MP and HP diets (*P* < 0.05) (Table 3).

**Table 3:** Bone mineral density (BMD) measures of the mid‐diaphysis of humerus and the mid‐diaphysis of femur, and the proximal, medial and distal parts of the metacarpal and metatarsal bones. BMD is shown as an overall total measure and for the trabecular and cortical part of the bone

		Diet^a^				*P*‐value
	BMD	LP	MP	HP	SEM	Diet
Humerus, medial, mg cm^−3^	Trabecular	79^a^	77^a^	53^b^	7.8	*
	Cortical	880^a^	1014^b^	1025^b^	19.3	***
	Overall	450^a^	584^b^	613^b^	34.5	*
Femur, medial, mg cm^−3^	Trabecular	55	65	56	5.2	NS
	Cortical	885^a^	1036^b^	1055^b^	10.3	***
	Overall	367^a^	597^b^	600^b^	24.3	***
Metacarpal:						
Proximal, mg cm^−3^	Trabecular	197	180	215	9.2	NS
	Cortical	528^a^	587^b^	561^c^	6.4	***
	Overall	275^a^	379^b^	353^c^	4.8	***
Medial, mg cm^−3^	Trabecular	31	30	37	3.9	NS
	Cortical	740^a^	856^b^	871^b^	11.0	***
	Overall	251^a^	375^b^	369^b^	6.9	***
Distal, mg cm^−3^	Trabecular	276^a^	302^b^	302^b^	2.4	***
	Cortical	475^a^	554^b^	556^b^	7.2	***
	Overall	301^a^	450^b^	456^b^	9.8	***
Metatarsal:						
Proximal, mg cm^−3^	Trabecular	161^ab^	191^a^	131^b^	14.2	*
	Cortical	563^a^	644^b^	629^b^	18.6	*
	Overall	218^a^	305^b^	300^b^	9.2	***
Medial, mg cm^−3^	Trabecular	56	48	65	15.7	NS
	Cortical	825	887	919	25.3	NS
	Overall	232^a^	344^b^	343^b^	13.8	***
Distal, mg cm^−3^	Trabecular	259^a^	314^b^	298^ab^	13.6	*
	Cortical	509	567	539	23.4	NS
	Overall	275^a^	422^b^	412^b^	11.7	***

a LP, low‐phosphorus diet; MP, medium‐phosphorus diet; HP, high‐phosphorus diet.

* *P* < 0.05,

*** *P* < 0.001.

^a,b,c^Values within a row with unlike lower‐case superscript letters were significantly different.

NS, not significant.

### Ash, Ca and P contents of bone

Ash, Ca and P contents of the bones were lower in pigs fed the LP diet regardless of bone and bone section (*P* < 0.01) (Table 4). However, the lower mineral content in the LP group was consistently more pronounced in the femur (e.g. ash: 16% lower in medial femur *versus* 10% lower in medial humerus compared to MP results). Moreover, the ash, Ca and P contents in the proximal and distal sections were markedly lower than in the medial section when the dietary P supply was low. In addition, this low mineral content was generally more pronounced in the proximal part than in the distal part of the bone (e.g. P in femur: 28% lower in the distal section *versus* 25% lower in the proximal section compared to MP results). No statistical differences in ash, Ca and P were found between pigs fed the MP and HP diets, but all results for humerus were numerically higher in pigs fed the HP diet compared with those fed the MP diet, while all femur results were numerically lower in pigs fed the HP diet than pigs fed the MP diet. The Ca:P ratios measured in the humerus and femur were higher when the pigs were fed the LP diet compared with MP and HP diets (*P* < 0.001), but no significant difference was seen between the MP‐ and HP‐fed pigs (Table 5).

**Table 4:** Ash and mineral measures of the proximal, medial and distal parts of humerus and femur

		Diet^a^				*P*‐value
		LP	MP	HP	SEM	Diet
Humerus:						
Ash, g kg^−1^ DM^b^	Proximal	33.97^a^	45.64^b^	48.47^b^	1.57	***
	Medial	55.73^a^	62.02^b^	64.71^b^	1.21	***
	Distal	43.73^a^	55.16^b^	55.17^b^	1.38	***
Phosphorus, g kg^−1^ DM	Proximal	5.69^a^	8.00^b^	8.65^b^	0.29	***
	Medial	9.47^a^	10.96^b^	11.40^b^	0.22	***
	Distal	7.26^a^	9.67^b^	9.70^b^	0.24	***
Calcium, g kg^−1^ DM	Proximal	12.43^a^	16.70^b^	17.00^b^	0.58	***
	Medial	21.00^a^	23.21^b^	24.10^b^	0.47	**
	Distal	16.07^a^	20.41^b^	20.39^b^	0.50	***
Femur:						
Ash, g kg^−1^ DM^b^	Proximal	37.96^a^	53.44^b^	52.92^b^	0.47	***
	Medial	49.99^a^	59.69^b^	58.14^b^	1.62	**
	Distal	37.43^a^	49.66^b^	48.55^b^	0.61	***
Phosphorus, g kg^−1^ DM	Proximal	6.35^a^	9.44^b^	9.37^b^	0.10	***
	Medial	8.50^a^	10.44^b^	10.21^b^	0.24	***
	Distal	6.26^a^	8.73^b^	8.46^b^	0.10	***
Calcium, g kg^−1^ DM	Proximal	13.89^a^	19.75^b^	19.55^b^	0.19	***
	Medial	18.88^a^	22.13^b^	21.47^b^	0.54	**
	Distal	13.75^a^	18.24^b^	17.63^b^	0.23	***

a LP, low‐phosphorus diet; MP, medium‐phosphorus diet; HP, high‐phosphorus diet.

b DM, dry matter.

^*^ *P* < 0.05,

** *P* < 0.01,

*** *P* < 0.001.

^a,b^Values within a row with unlike lower‐case superscript letters were significantly different.

**Table 5:** Calcium:phosphorus ratio in three sections of the humerus and femur

	Diet^a^				*P*‐value
	LP	MP	HP	SEM	Diet
Humerus:					
Proximal	2.19^a^	2.09^b^	2.08^b^	0.008	***
Medial	2.22^a^	2.12^b^	2.11^b^	0.004	***
Distal	2.22^a^	2.11^b^	2.10^b^	0.007	***
Femur:					
Proximal	2.19^a^	2.09^b^	2.09^b^	0.008	***
Medial	2.22^a^	2.12^b^	2.10^b^	0.006	***
Distal	2.20^a^	2.09^b^	2.08^b^	0.008	***

a LP, low‐phosphorus diet; MP, medium‐phosphorus diet; HP, high‐phosphorus diet.

*** *P* < 0.001.

^a,b^Values within a row with unlike lower‐case superscript letters were significantly different.

## DISCUSSION

Generally, the observed low bone net mineralisation in pigs fed the LP diet is in agreement with other studies confirming that deficient P supply leads to reduced mineral content of bones.^9^, ^10^, ^13^, ^14^, ^25^, ^26^, ^27^

Similar to previous reports,^13^, ^25^ the present results showed that the LP supply was not sufficient to achieve the same overall BMD level in the diaphysis of all four studied bones as the MP and HP supplies. This is supported by previous results showing that decreased P supply to pigs reduced body P retention.^20^ Furthermore, the lower overall BMD caused by LP was more pronounced in the femur than in the humerus. In addition, the LP‐induced lower ash, Ca and P contents were more distinct in the femur than in the humerus suggesting a larger metabolic activity in the femur. This negative influence on bone mineralisation is supported by data showing no significant difference in wet weight of humerus and femur with increased P supply; however, low P supply caused lower dry weight of the two bones (*P* < 0.01).^20^ Farries *et al.* 28 found that reproducing sows were more likely to mobilise minerals from the bones of the hind legs than from other bones which is supported by the present results. Furthermore, the overall BMD observed in pigs fed the LP diet was equally low in the medial metacarpal and metatarsal but to a larger extent than in the humerus. However, the overall BMD was in general lower in all three sections of the metatarsal than of the metacarpal. These observations challenge the statement by Farries *et al.* 28 who indicated that the effects of varied dietary P cannot exclusively be addressed to the front or hind legs even though femur was affected the most by the LP diet in the present study. It can be speculated that some bones are more prone to metabolic activity than others because of anatomic localisation and function, e.g. some bones being more weight‐bearing or large enough to serve as a reserve of minerals. Jointly, Thorup *et al.* 29 who found that the front and hind legs differ biomechanically as the front legs carry more weight than the hind legs, and Ryan *et al.* 30 who showed that the front legs had higher BMD than the hind legs support this interpretation. Furthermore, findings in small children show that the femur strength increases rapidly at the time when they start to walk, whereas at the same time the increase in the strength of the humerus slows down.^31^ Farries *et al.* 28 refer to mobilisation of minerals in order to fulfil the demands for foetus growth and milk production in sows. However, these mechanisms can probably be modified to also include mineralisation of the growing bone, since the same bones most likely are involved in both mineralisation and demineralisation. Baylink *et al.* 32 indicated that severely low dietary P (4 g kg^−1^ in test diet *versus* 6 g kg^−1^ in control diet) fed to rats caused a decreased bone matrix formation rate and consequently a delayed onset of mineralisation of the osteoid. It is therefore suggested that the low BMD, ash, Ca and P values obtained in the present study due to the long‐term low dietary P supply are results of reduced bone formation rather than a massive resorption because the mineralisation process is dominant during growth.^33^

In the present study, an LP‐induced lower trabecular BMD was solely found in the distal section of the metacarpal, which indicates that the distal trabecular tissue is more involved in bone mineral metabolism than the proximal part. Buhler *et al.* 25 found a similar reduction in the trabecular BMD in pigs weighing 66 kg when dietary P decreased even though the difference was counterbalanced at 108 kg BW (1.4 *versus* 2.6 g digestible P kg^−1^). Moreover, Larsen *et al.* 26 found that low dietary Ca (3.5 *versus* 9.4 g kg^−1^ DM) resulted in a 36% lower trabecular bone volume and increased osteoid thickness. Furthermore, Eklou‐Kalonji *et al.* 10 showed reduced bone formation rate when the Ca supply was low (1.1–3.8 *versus* 9.0 g kg^−1^ diet). No BMD measures were performed in the proximal and distal femur (lack of resources), but it is assumed that trabecular BMD will also be lower, since femur tissue was found to be more prone to dietary P deficiency in the present study. In comparison, the ash, Ca and P contents of the proximal and distal bones were all lower in pigs fed the LP diet, and, as such, these results indicate if either of the two sections is more metabolically active. Pond *et al.* 34 showed that ^45^Ca injection to growing pigs resulted in the highest activity in the proximal and distal sections of the humerus and femur. Those authors found that the distal section of the femur showed the highest activity of ^45^Ca and was the most metabolically active site. This is in line with the present results. Pond *et al.* 34 also discovered that the nasal turbinate and the vertebra also showed high ^45^Ca activity whereas teeth showed very low activity. These results indicate that various bones and bone segments are metabolically active but mineralise differently.

The present study showed that the diaphysis of the bones contained the largest concentrations of ash, Ca and P, and that the LP diet resulted in a smaller reduction in the diaphysis than in the proximal and distal parts. This supports earlier findings of trabecular tissue being more involved in mineral exchange than the cortical tissue^10^, ^27^, ^34^ and has more capacity to contribute to bone metabolism because of a relatively larger surface area compared with the cortical tissue.^35^ It can be speculated that the longitudinal strength and stability of the diaphysis (mainly cortical tissue) are favoured when the P supply is deficient. This may be crucial in order to ensure the function of the skeleton.

The present results indicate that the bone Ca:P ratio can be manipulated through different dietary P supply. These results confirm previous reports showing that the Ca:P ratio is increased in the femur but not in the tibia–fibula in pigs,^14^, ^36^ whereas the Ca:P ratio in the scapula in growing‐finishing pigs was 2.15 regardless of the level of Ca and P supply.^37^ Overall, it is evident that bones are differently involved in the exchange of minerals during depletion of Ca and P.^9^, ^28^, ^34^ The reason for this difference may be that when Ca and/or P supply is limited, the body must prioritise the most critical bones for optimal skeletal function, and therefore the demineralisation (or reduced mineralisation) of the skeleton is initiated in bones that are less vulnerable to functional damage when the mineral supply is reduced.

No major differences were found between the different bone sections between the MP‐ and the HP‐fed pigs, which confirmed that the MP level was sufficient for maintaining net bone mineralisation in the growing‐finishing period. However, most interestingly the total contents of ash, Ca and P in pigs fed HP were numerically increased in the humerus but lowered in the femur. Maxson and Mahan^27^ reported that an increase in the dietary Ca and P levels caused a relatively greater increase in net mineralisation, especially in the metaphyseal sections of the humerus and femur. The present results may also indicate that the proximal and medial sections of the humerus are able to deposit surplus minerals whereas the distal section is not. This means that an increase in dietary P may lead to increased net mineralisation of the humerus but not of the femur. This is yet another indication of different bones having different capacities to store minerals, and the difference between the humerus and femur is in line with the femur participating more in the mineral exchange than the humerus. Fernandez^5^ found comparable levels of Ca and P retention in pigs fed diets corresponding to the present levels of the HP and MP diets. At the same time, Fernandez^5^ also concluded that an increased bone mineral accretion is not necessarily beneficial, since it may increase the stiffness and brittleness of the bone material and thereby decrease the ability of bone to absorb energy without breaking.^8^, ^16^, ^38^ This indicates that excessive P supply may be harmful to overall bone function.

BMD, ash, Ca and P measures all have a common purpose: to give indications about the strength of a bone. Nielsen *et al.* 38 demonstrated a positive correlation between BMD and bone strength in finishing pigs, and Liesegang *et al.* 13 found that a P‐deficient diet caused reduced BMD and bone ash in the femur and tibia of pigs, and that these two parameters were highly correlated. Furthermore, Eklou‐Kalonji *et al.* 10 showed that bone strength was low in Ca‐deficient pigs, and that bone strength was positively correlated with bone mineral content indicating that BMD, ash, Ca and P all together have major influence on bone strength. The present results to some extent comply with previous results since the relationship between BMD and ash, Ca and P only tended to correlate. However, the present study is interesting because it includes studies on both front and hind legs, which substantiates the overall conclusion that bones are not equally optimal for determination of strength and adequate P supply. As such, the results are valuable for future studies of reduction of P emissions in pig farming involving pig health and welfare along with performance and emissions. The MP level in the present study reveals a safe level of P in contrast to the LP level of P. However, it does not uncover if it is the lowest safe level of P. Therefore, this level may be a starting point for designing future studies.

## CONCLUSIONS

Feeding a P‐deficient diet during the entire growing‐finishing period resulted in a low net mineralisation of the humerus, femur, metacarpal and metatarsal bones in pigs, revealing femur being the most affected and metabolically active bone. However, no significant additional bone mineral retention was found when supplying the pigs with excessive amounts of P above requirement, though indications of an increased net mineralisation of the humerus were found. In general, the diet deficient in P (LP diet) caused low BMD, ash, Ca and P which testifies reduced bone strength. The trabecular tissue was shown to be noticeably more negatively affected than the cortical tissue. In conclusion, the MP level (6.2 g P kg^−1^ DM equal to 3.13 g digested P kg^−1^ DM) is sufficient for maintaining bone net mineralisation in growing‐finishing pigs, because no increase in BMD, ash, Ca and P was seen when pigs were fed increased dietary P (HP: 8.9 g P kg^−1^ DM equal to 4.9 g digested P kg^−1^ DM). Furthermore, it cannot be concluded whether the dietary P content can be slightly reduced beyond the MP level without negative effects. Femur is recommended as the target bone for studying bone fragility whereas the humerus seems to be useful for studying surplus P retention in order to reduce P emissions in a safe and balanced manner in pig farming involving aspects of pig health and welfare along with performance and emissions.

## CONFLICT OF INTEREST

None.

## IMPLICATIONS

Phosphorus should be fed in adequate amounts to sustain body development and bone mineralisation and oversupply should be avoided due to environmental reasons. Further, P supplies below or above requirement may also result in lowered performance, e.g. the weight gain of pigs. In order to fine‐tune a proper P supply ensuring pig health, welfare, performance and environmental aspects, there is a demand for knowledge on the interplay between P supply and physiology. The viewpoint is to develop more individual P feeding standards addressing that different pig production systems, breeds, etc., have different growth rates and feed intake, and thereby also different daily P requirements. This study shows which bones to be used as target points for evaluation of mineralisation in growing pigs.
